# Menthol facilitates the intravenous self-administration of nicotine in rats

**DOI:** 10.3389/fnbeh.2014.00437

**Published:** 2014-12-16

**Authors:** Tengfei Wang, Bin Wang, Hao Chen

**Affiliations:** ^1^Department of Pharmacology, University of Tennessee Health Science CenterMemphis, TN, USA; ^2^College of Pharmacy, Shaanxi University of Chinese MedicineXian Yang, Shaanxi, China

**Keywords:** nicotine, menthol, self-administration, rats, sensory cue

## Abstract

Menthol is preferred by ~25% of smokers and is the most common flavoring additive in tobacco and electronic cigarettes. Although some clinical studies have suggested that menthol facilitates the initiation of smoking and enhances the dependence on nicotine, many controversies remain. Using licking as the operant behavior, we found that adolescent rats self-administering nicotine (30μg/kg/infusion, free base, i.v.) with contingent oral menthol (60μl, 0.01% w/v) obtained significantly more infusions than rats receiving a vehicle cue or rats self-administering i.v. saline with a menthol cue. Rats yoked to their menthol-nicotine masters emitted significantly fewer licks on the active spouts, indicating that contingent pairing between nicotine and menthol is required for sustained nicotine intake. Rats that self-administered nicotine with a menthol cue also exhibited a long-lasting extinction burst and robust reinstatement behavior, neither of which were observed in rats that self-administered saline with a menthol cue. The cooling sensation of menthol is induced by activating the transient receptor potential M8 (TRPM8) channel. When WS-23, an odorless agonist of the TRPM8 channel, was used as a contingent cue for nicotine, the rats obtained a similar number of nicotine infusions as the rats that were provided a menthol cue and exhibited a strong preference for the active spout. In contrast, highly appetitive taste and odor cues failed to support nicotine self-administration. These data indicated that menthol, likely by inducing a cooling sensation, becomes a potent conditioned reinforcer when it is contingently delivered with nicotine. Together, these results provide a key behavioral mechanism by which menthol promotes the use of tobacco products or electronic cigarettes.

## 1. Introduction

Menthol is the most widely used tobacco additive and is preferred by ~25% of US smokers (Giovino et al., 2004). Smokers who prefer menthol cigarettes are more likely to be female, young, and less educated (Fernander et al., 2010; Lawrence et al., 2010). Compared with Caucasian smokers, significantly more African American smokers (70%) prefer menthol cigarettes (Lawrence et al., 2010; Trinidad et al., 2010). Additionally, menthol is one of the most preferred flavors of electronic cigarettes (McQueen et al., 2011). Although there has been a steady decrease in cigarette smoking, the use of electronic cigarettes is increasing at an alarming rate, with the use among US teenagers doubling from 2011 to 2012 (Center for Disease Control and Prevention, 2013). Therefore, understanding the interaction between the sensory properties of menthol and the reinforcing effect of nicotine is urgently required.

Many studies have investigated the effect of menthol on smoking behavior; some reported that menthol facilitated the initiation of smoking and enhanced the dependence on nicotine (Hersey et al., 2006; Muscat et al., 2012; Nonnemaker et al., 2013), whereas others reported that smoking menthol cigarettes was associated with a lower responsiveness to medication, greater difficulty in quitting, and higher likelihood of relapse (Pletcher et al., 2006; Gundersen et al., 2009; Cubbin et al., 2010; Stahre et al., 2010; Levy et al., 2011). However, many other studies have failed to identify a significant effect of menthol [see review by Hoffman and Simmons (2011)]. These inconsistencies are likely caused by differences in the study populations, study designs, and difficult-to-control environmental and socioeconomic factors.

Another potential source for the inconsistencies in the literature is the complex pharmacological and affective effects of nicotine. For example, nicotine serves as a strong reinforcer of operant behaviors in a wide range of species, from rodents (Corrigall and Coen, 1989; Chen et al., 2007) to humans (Rose et al., 2003). Furthermore, nicotine also induces strong aversive responses (Fowler et al., 2011; Fowler and Kenny, 2013). We recently showed that the sensory modality of the cue associated with nicotine delivery has a strong role in determining the overall affective value of nicotine. In particular, we reported that olfactogustatory cues were associated with the aversive effect of self-administered nicotine (Chen et al., 2011). Furthermore, genetic studies have consistently shown that the gene cluster on chromosome 15 that encodes nicotinic acetylcholine receptor subunits α5, α3 and β4, which underlies the aversive response to nicotine (Fowler and Kenny, 2013), is associated with cigarette smoking. Therefore, the interactions between the complex sensory stimulation elicited by menthol and the pharmacological effect of nicotine could be responsible for some of the inconsistencies found in clinical studies.

Menthol elicits complex multimodal sensory stimulation, including a strong cooling sensation mediated by the transient receptor potential M8 (TRPM8) channel (Voets et al., 2004), a slightly bitter taste detected mainly in the circumvallate region of the tongue (Green and Schullery, 2003), and a strong minty odor. Additionally, menthol has an analgesic effect that is mediated by the transient receptor potential A1 channel (Macpherson et al., 2006).

In this study, we report the effect of orally delivered menthol as a cue for the intravenous self-administration (IVSA) of nicotine in adolescent rats, using licking as the operant response (Levin et al., 2010; Chen et al., 2011). We first compared the effects of menthol and vehicle cues on nicotine self-administration. Because menthol induces a multimodal sensory stimulation, we subsequently investigated the effects of the cooling sensation and olfactogustatory cues. The interaction between menthol and an audiovisual cue was also studied. Finally, we investigated the effect of menthol on extinction and reinstatement. Overall, our data showed that the cooling sensation of menthol facilitated the intake of nicotine.

## 2. Materials and methods

### 2.1. Animals

Sprague-Dawley rats were purchased from Harlan Laboratories (Madison, WI). Female rats were used because menthol cigarette smokers are more likely to be female (Fernander et al., 2010; Lawrence et al., 2010). Similar to our previous studies (Chen et al., 2007, 2012), the rats arrived at our animal facility on approximately postnatal day 31 and received surgery on approximately postnatal day 38. The definition of adolescence in rodents is controversial. According to a conservative perspective on rodents (Spear, 2000), prototypical adolescent changes occur during postnatal days 28–42. Some developmental changes specific to adolescence do persist through postnatal day 55 (Spear, 2000). Thus, the present experiments were performed within the broadly defined age range of adolescence. The rats were group housed in rooms with a reverse light cycle (lights on from 9:00 pm to 9:00 am). Standard rat chow and water were provided *ad libitum*. All procedures followed the NIH Guidelines Concerning the Care and Use of Laboratory Animals and were approved by the Animal Care and Use Committee of the University of Tennessee.

### 2.2. Intravenous nicotine self-administration

A sterile Micro-Renathane catheter (OD = 0.94 mm, ID = 0.58 mm, Braintree Scientific Inc., Braintree, MA) was implanted on approximately postnatal day 38 (Chen et al., 2007). The catheter exited from the back of the animal through a polylactic acid implant (described in detail below). Pain medication (Ketofen, 2 mg/kg) was provided immediately after surgery.

The rats received no prior operant training, water or food deprivation, or social isolation before IVSA; they were group housed according to the treatment conditions throughout the experiment. Daily 3-h nicotine IVSA sessions were conducted in operant chambers (Med Associates Inc., St. Albans, VT) equipped with two stainless steel sipping tubes containing metal ball bearings. Both sipping tubes were connected to contact lickometer controllers. Syringes containing nicotine (30μg/kg/injection, free base, pH 7.0–7.4) and flavor cues were filled prior to each session. Polyethylene tubing connected the nicotine syringe to the jugular catheter through a swivel; the flavor cue was delivered near the metal ball bearings through polyethylene tubing. The flavored solutions included menthol (0.01% w/v, in 0.01% Tween 80), vehicle (0.01% Tween 80), a mixture of saccharin (0.125%) and glucose (3%), and unsweetened grape-flavored Kool-Aid® (0.1%). WS-23 (2-isopropyl-N,2,3-trimethylbutyramide; The Ingredient House, Pinehurst, NC. 0.01% and 0.03% in water), which is a TRPM8 channel agonist that has a potency within the same order of magnitude as menthol (Behrendt et al., 2004), was also used as a sensory cue. One additional group received a composite cue of 0.01% WS-23, 0.4% saccharin, and 0.1% Kool-Aid®.

Licking on the active spout meeting a fixed-ratio 10 reinforcement schedule with a 20 s timeout simultaneously activated both syringe pumps (model PHM100 with a 15-rpm motor, Med Associates) to deliver nicotine or saline in 0.8–1.2 s based on body weight and 60 μl flavor cue in 0.76 s. Therefore, licking on the active spout (hereafter referred to as active licks) delivered the flavor cue only when nicotine was also delivered. Licking on the inactive spout (i.e., inactive licks) had no programmed consequence. A contingent audiovisual cue was provided for three additional groups (oral vehicle with i.v. nicotine, oral menthol with i.v. saline, and oral menthol with i.v. nicotine). The audiovisual cue consisted of the onset of a tone generator (Malloary Sonalert, SC628, 80 dB) and a white cue light (Med Associates, ENV-221M, with 100 mA bulb) located above the spouts for the duration of the nicotine infusion. For the yoked control experiment, each yoked rat was paired with a master rat but was placed in its own operant chamber. The yoked rats received a nicotine infusion concurrently with their master's self-administered nicotine. When the yoked rats licked the active spout, they received menthol but not nicotine.

A stock menthol solution (Sigma, St. Louis, MO) (10×, 0.1% menthol, and 0.1% Tween 80) was heated (25 s) in a microwave oven, mixed, and maintained at room temperature for a maximum of 7 days. A fresh menthol working solution (1×) was prepared daily. This concentration of menthol was used because it can unequivocally activate the thermal-sensitive lingual fibers (Lundy and Contreras, 1994). Methohexital (5 mg/kg; JHP Pharmaceuticals, Rochester, MI), a fast-acting anesthetic, was used to verify the patency of the jugular catheters. Rats that failed this test were excluded from the analysis.

### 2.3. Cold water as a sensory cue for nicotine IVSA

One group of rats self-administered nicotine with contingent cold water (60 μl). Several modifications were made to the cue delivery setup to ensure that the water was delivered at a constant low temperature throughout the 3-h IVSA sessions. The end of the stainless steel spout located outside of the operant chamber was sealed and placed in a bottle filled with a nontoxic cooling gel. These bottles were stored at −80°C before use. Water delivered by syringe pumps first entered a ring of plastic tubing (55 cm, ID = 0.76 mm) submerged in ice water. The plastic tubing was then connected to stainless steel tubing that entered a small opening 2 cm from the tip of the spout located inside the operant chamber. Cold water was delivered close to the ball bearing inside the spout. The water temperatures measured at the tip of the drinking spout were 9.3 ± 0.3, 11.2 ± 0.3, 11.3 ± 0.3, and 11.9 ± 0.4°C at 0, 1, 2, and 3 h, respectively. The room temperature was 18.7–20.2°C during the experiment. A picture of the setup is presented in Figure S1.

### 2.4. Extinction and reinstatement of nicotine seeking

During the 3-h extinction sessions, licking was recorded but had no programmed consequence. Rats met the extinction criteria when the number of licks on the active spout were less than 150 (i.e., ~20% of those on the first day of extinction in the menthol-nicotine group) for two consecutive days. The reinstatement of nicotine seeking was then tested. During the 3-h reinstatement sessions, active spout licking delivered a menthol solution (60 μl) under a fixed-ratio 10 schedule with a 20 s timeout. Nicotine was not delivered.

### 2.5. Manufacturing the surgical implant using 3D printing

A MakerBot® Replicator 2 (Makerbot Industries, Brooklyn, NY) was used to manufacture the implant from polylactic acid. The implant was printed in 0.1 mm layers. Stainless steel tubing (4 mm, 23 gauge) was inserted 15 mm into the distal end of a 12 cm Micro-Renathane tube to provide a strong surface for tying sutures over the jugular vein. The proximal end of this tubing was connected to a 35 mm, 23 gauge stainless steel tube, which was then inserted into the center of the implant and extended 5 mm beyond the implant. One 10 mm to 12 mm stainless steel spring was placed outside the stem of the implant to prevent it from being damaged during group housing. The implant design and an image of the assembled implant are presented in Figure S2 (see also http://www.thingiverse.com/haochen/).

### 2.6. Odor habituation test

One perforated divider was placed in the middle of the operant chamber with all operant manipulanda removed. The rats and odorants were placed on opposite sides of the divider. Menthol (0.01%, dissolved in 0.01% Tween 80), WS-23 (0.01% or 0.03% in water), or water (1 mL each) was placed in a plastic weighing boat on the chamber floor ~1 cm from the divider. Eleven naive female Sprague-Dawley rats were tested. Each rat was tested with all three odorants daily for two consecutive days. The odorants were always placed in the same test chambers to avoid potential odor contamination. The odorant sequence was counterbalanced among the rats. The number of nose pokes into the divider was recorded for 20 min by infrared sensors embedded in the divider. The rats remained in their home cages for > 1 h between tests.

### 2.7. Lick microstructure analysis

The timing of licks on the active spout was analyzed for its microstructure. Licks with interlick intervals of less than 0.5 s were treated as one cluster. Clusters with less than two licks were excluded from the analysis. The size of the lick cluster, defined as the number of licks contained within a cluster, corresponded to the appetitive nature of the solution (Davis and Smith, 1992).

### 2.8. Statistical analysis

The number of licks and the ratio of active/inactive licks were log-transformed to fit a normal distribution prior to statistical analysis. The data were presented as the mean ± SE. Repeated-measures analysis of variance (ANOVA) was used to analyze the number of licks and infusions, with the session and spout treated as within-subject variables and treatment groups, including the cue used (e.g., menthol, vehicle, WS-23, etc.) and i.v. solution (i.e., nicotine or saline), as between-subject variables. Because the number of active licks included those occurred during the 20 s time-out period, which had no behavioral consequence, these data were noisier than the number of infusions. Therefore, with the exception of the yoke experiment, we, in general, analyzed the number of nicotine infusions when different experimental conditions were compared. The number of active licks were compared between the yoked and the the master groups because the number of infusions was the same between these two groups by design. Tukey's HSD test was used for *post-hoc* analysis when appropriate. All analyses were conducted using the R statistical analysis package.

## 3. Results

### 3.1. Oral menthol cue supports increasing i.v. nicotine intake

**Figure 1:**
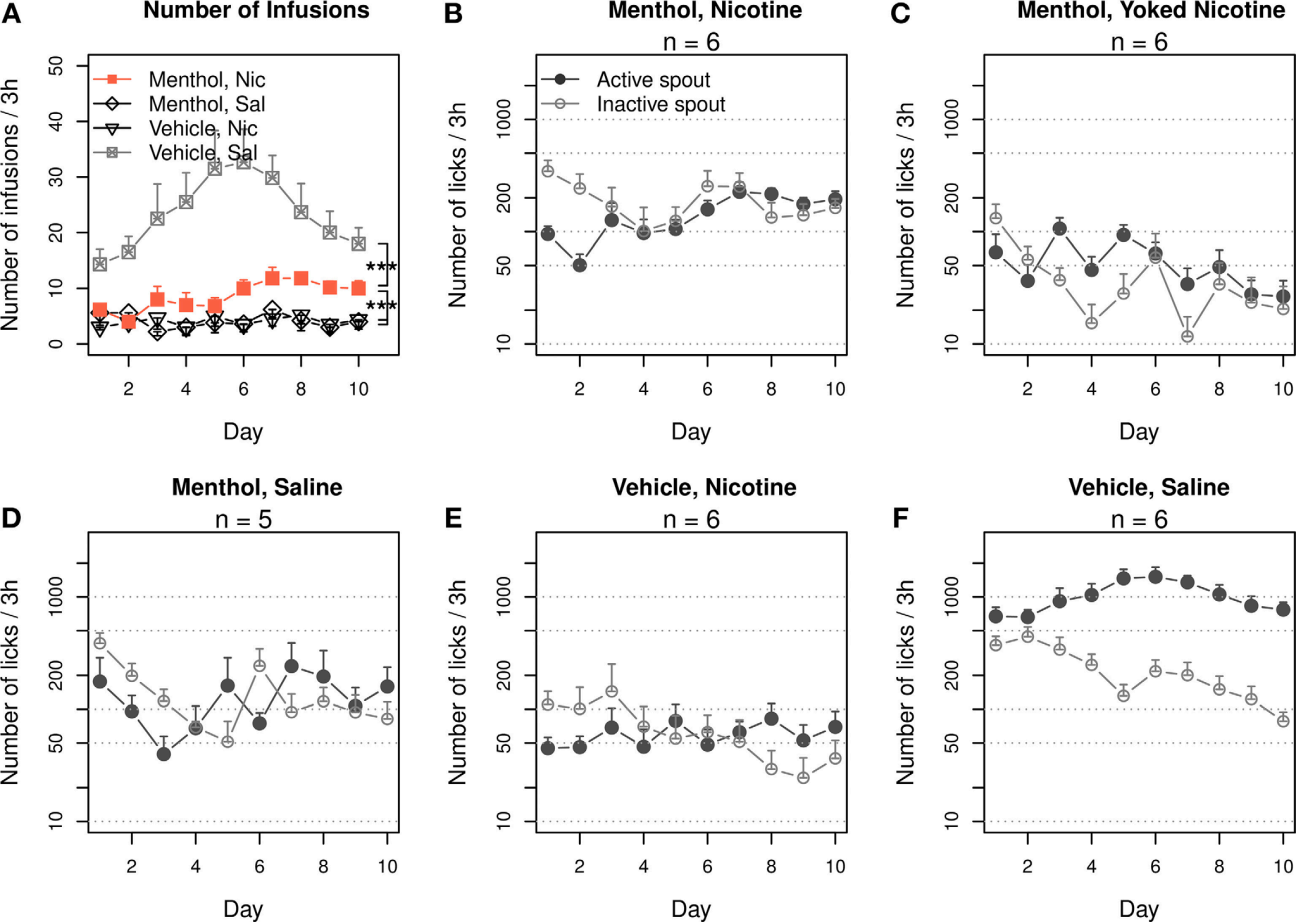
**Contingent oral menthol supports stable i.v. nicotine self-administration. (A)** Female adolescent Sprague-Dawley rats received concurrent oral menthol (or vehicle) cue and i.v. nicotine (or saline) upon the completion of a fixed-ratio 10 reinforcement schedule on the lickometer. The number of infusions obtained per session by the menthol-nicotine group was significantly greater than that obtained by the menthol-saline and vehicle-nicotine groups, indicating that the contingent delivery of menthol and nicotine is critical for increased intake. **(B–F)** The numbers of active and inactive licks by all four treatment groups and one additional group of rats yoked to the menthol-nicotine group. A log scale was used for the y-axis. Only the menthol-nicotine group significantly increased the number of active licks and sustained the level of responses across the sessions, confirming the reinforcing effect of the menthol-nicotine stimuli. With the exception of the vehicle-saline group, none of the groups exhibited a preference for the active spout, suggesting that despite being reinforcing, neither menthol nor nicotine produced a positive affective state (see **Figure 6**). ^***^ *p* < 0.001.

The rats were trained to self-administer i.v. nicotine with a contingent oral menthol cue. The control groups self-administered i.v. nicotine with a contingent vehicle cue, i.v. saline with menthol cue, or i.v. saline with vehicle cue. The numbers of infusions that these groups obtained are shown in Figure 1A. Repeated-measures ANOVA found significant main effects by session (F_9, 171_ = 3.1, *p* < 0.01), nicotine (F_1, 19_ = 23.0, *p* < 0.001), and menthol (F_1, 19_ = 15.4, *p* < 0.001). There was also a significant interaction between nicotine and menthol (F_1, 19_ = 26.8, *p < 0.001*). The number of infusions did not significantly change across the sessions in the menthol-saline (F_9, 36_ = 1.2, *p* > 0.05) or vehicle-nicotine (F_9, 45_ = 0.5, *p* > 0.05) groups. On average, these control rats obtained < 5 infusions per session. The number of infusions in the vehicle-saline group significantly changed during the 10 daily sessions (F_9, 45_ = 2.6, *p* < 0.05), peaking in the sixth session (32.6 ± 5.9 infusions) and decreasing to 18.0 ± 2.9 infusions during the tenth session. The rats in the menthol-nicotine group significantly increased the number of infusions (F_9, 45_ = 3.3, *p* < 0.01) from 6.2 ± 1.0 infusions during the first session 1 to 10.0 ± 1.5 during the sixth session, and the number of infusions remained greater than 10 thereafter. Thus, the vehicle-saline group obtained a significantly greater number of infusions than the menthol-nicotine group (F_1, 10_ = 23.5, *p* < 0.001), menthol-saline group (F_1,9_ = 32.4, *p* < 0.001), and the vehicle-nicotine group (F_1, 10_ = 39.0, *p* < 0.001), suggesting that both menthol and nicotine limited the number of infusions. However, the number of infusions obtained by the menthol-nicotine group was significantly greater than that obtained by the menthol-saline (F_1,9_ = 12.0, *p* < 0.01) and vehicle-nicotine control groups (F_1, 10_ = 13.2, *p* < 0.01), indicating that contingent presentations of menthol with nicotine enhanced the reinforcing effect of nicotine.

Figures 1B,D–F show the numbers of active and inactive licks by each group. We transformed the numbers of licks to a logarithmic scale to fit a normal distribution. The gradual increase in nicotine intake (Figure 1A) in the menthol-nicotine group was driven by the significant increase in the number of licks on the active spout across the sessions (F_9, 45_ = 4.8, *p* < 0.001). In contrast, the group of rats yoked to these menthol-nicotine rats (Figure 1C) significantly reduced the number of licks on the active spout across the sessions (F_9, 45_ = 3.1, *p* < 0.01). Consequently, the yoked rats emitted significantly less active licks compared to their masters (F_1, 10_ = 18.1, *p* < 0.01). In agreement with Figure 1A, none of the control groups exhibited a significant change in the number of licks across the sessions (*p* > 0.05 for all). With the exception of the vehicle-saline group (F_1, 50_ = 174.3, *p < 0.001*), none of the other groups showed a preference for the active spout (*p* > 0.05 for all).

### 3.2. Appetitive oral taste and odor cues do not support i.v. nicotine intake

**Figure 2:**
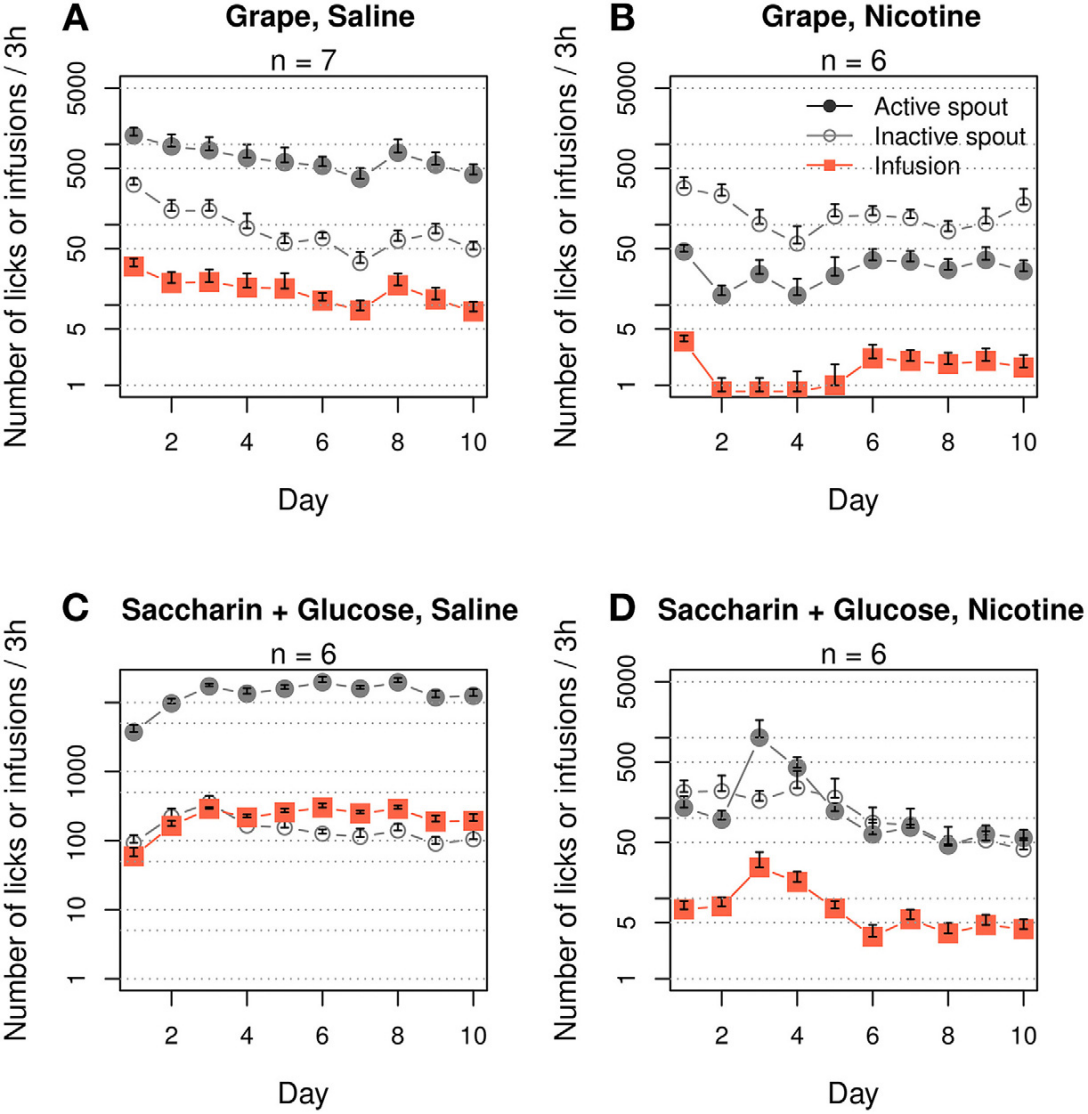
**Contingent appetitive oral cues did not support i.v. nicotine self-administration**. Female adolescent rats that self-administered saline with a contingent grape odor **(A)** or a saccharin and glucose mixture **(C)** exhibited a strong preference for the stimuli, suggesting they are both appetitive. However, neither of these cues supported nicotine (30 μg/kg/infusion) IVSA **(B** and **D)**. The number of nicotine infusions was ~5 on the majority of days and failed to increase across the 10 daily sessions.

Menthol induces a multimodal sensory stimulation, including strong odor and taste. We were unable to find a chemical that mimics the odor and taste of menthol that does not simultaneously induce a cooling sensation. Assuming that aversive taste or odor is unlikely to support nicotine intake, we examined the general effects of contingent appetitive odor and taste cues on nicotine IVSA. The rats exhibited a strong preference for the active spout when grape odor was paired with an i.v. saline infusion (Figure 2A, F_1, 60_ = 110.6, *p* < 0.001). On average, 15.8 ± 2.0 infusions were obtained during the 10 daily sessions (effect of session: F_9, 54_ = 1.5, *p* > 0.05). However, when grape odor was paired with i.v. nicotine infusions, the rats strongly avoided the active spout (Figure 2B, F_1, 50_ = 82.3, *p < 0.001*). On average, 1.7 ± 0.26 infusions were obtained during the 10 sessions (effect of session: F_9, 45_ = 1.5, *p* > 0.05).

We then tested a saccharin/glucose mixture, which incites highly appetitive behavior in rodents (Smith et al., 1976). The rats licked the active spout ~10,000 times after five sessions when i.v. saline was delivered (Figure 2C, effect of spout: F_1, 40_ = 466.0, *p* < 0.001). On average, the rats obtained 152.0 ± 23.3 infusions per session (effect of session: F_9, 36_ = 6.8, *p* < 0.001). However, the rats did not prefer the active spout when this solution was delivered contingently with nicotine (Figure 2D, F_1, 40_ = 2.5, *p > 0.05*). On average, the rats obtained 8.5 ± 2.1 infusions. The number of infusions peaked on session three (24.3 ± 13.4) and then significantly decreased (effect of session: F_9, 45_ = 2.1, *p < 0.05*) to 4.2 ± 0.2 for the last three sessions.

### 3.3. Oral cooling sensation supports i.v. nicotine intake

**Figure 3:**
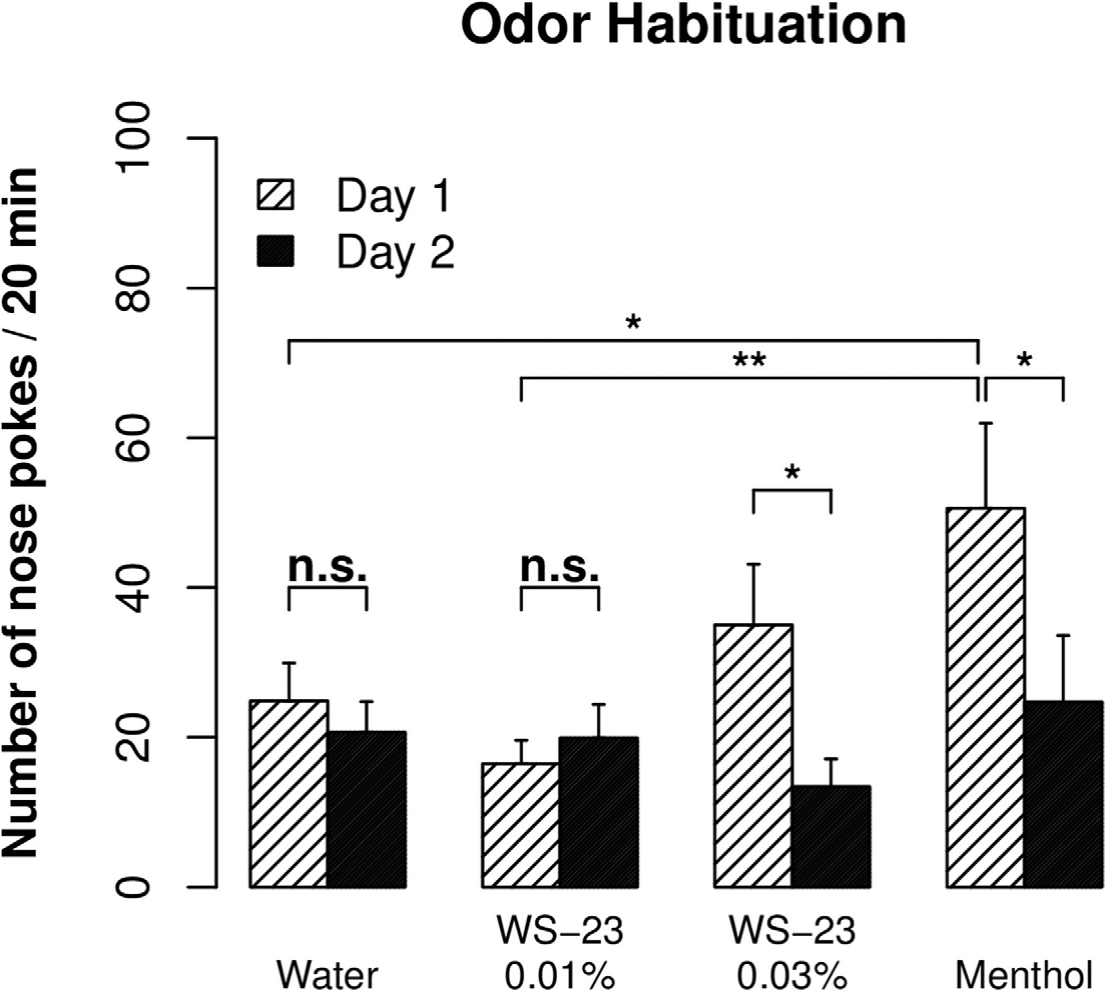
**The cooling compound WS-23 was odorless at low concentrations**. An odor habituation test was conducted for water, menthol (0.01%), and WS-23 (0.01 and 0.03%) over two consecutive days. Menthol and 0.03% WS-23 induced more nose pokes than water on day 1, and the number of nose pokes significantly decreased during the second test (i.e., habituation). In contrast, 0.01% WS-23 induced a similar number of nose pokes as water and there was no habituation, indicating that WS-23 is odorless. ^*^ *p* < 0.05, ^**^ *p* < 0.01.

Cooling, the prominent sensory property of menthol, is mediated by the TRPM8 channel (Voets et al., 2004). The WS-23 compound also stimulates the TRPM8 channel and has been reported to have virtually no taste or odor (Gaudin et al., 2008). We nevertheless used an odor habituation test (Inagaki et al., 2010) to examine whether WS-23 has an odor that can be detected by rats. There was a significant reduction in the number of nose pokes observed for 0.01% menthol from day 1 to day 2 (Figure 3, *p* < 0.01), reflecting habituation of the rats to the odor of menthol. In contrast, the number of nose pokes for water did not change between the two test sessions (*p* > 0.05). Furthermore, significantly fewer nose pokes were observed for water compared to menthol on day 1 (*p* < 0.05). These data established the validity of the assay.

The number of nose pokes for 0.03% WS-23 was significantly reduced between the two test sessions (*p* < 0.05). The number of nose pokes for 0.03% WS-23 was not different from that for menthol (*p* > 0.05). Although the number of nose pokes for 0.03% WS-23 was not significantly different from that for water (*p > 0.05)*, the overall data suggested that 0.03% WS-23 is likely to emit an odor that can be detected by rats. The number of nose pokes for 0.01% WS-23 was significantly lower than that for menthol (*p* < 0.01), not different from that for water (*p* > 0.05), and did not change between the two test sessions (*p* > 0.05). These data indicated that 0.01% WS-23 had no detectable odor.

**Figure 4:**
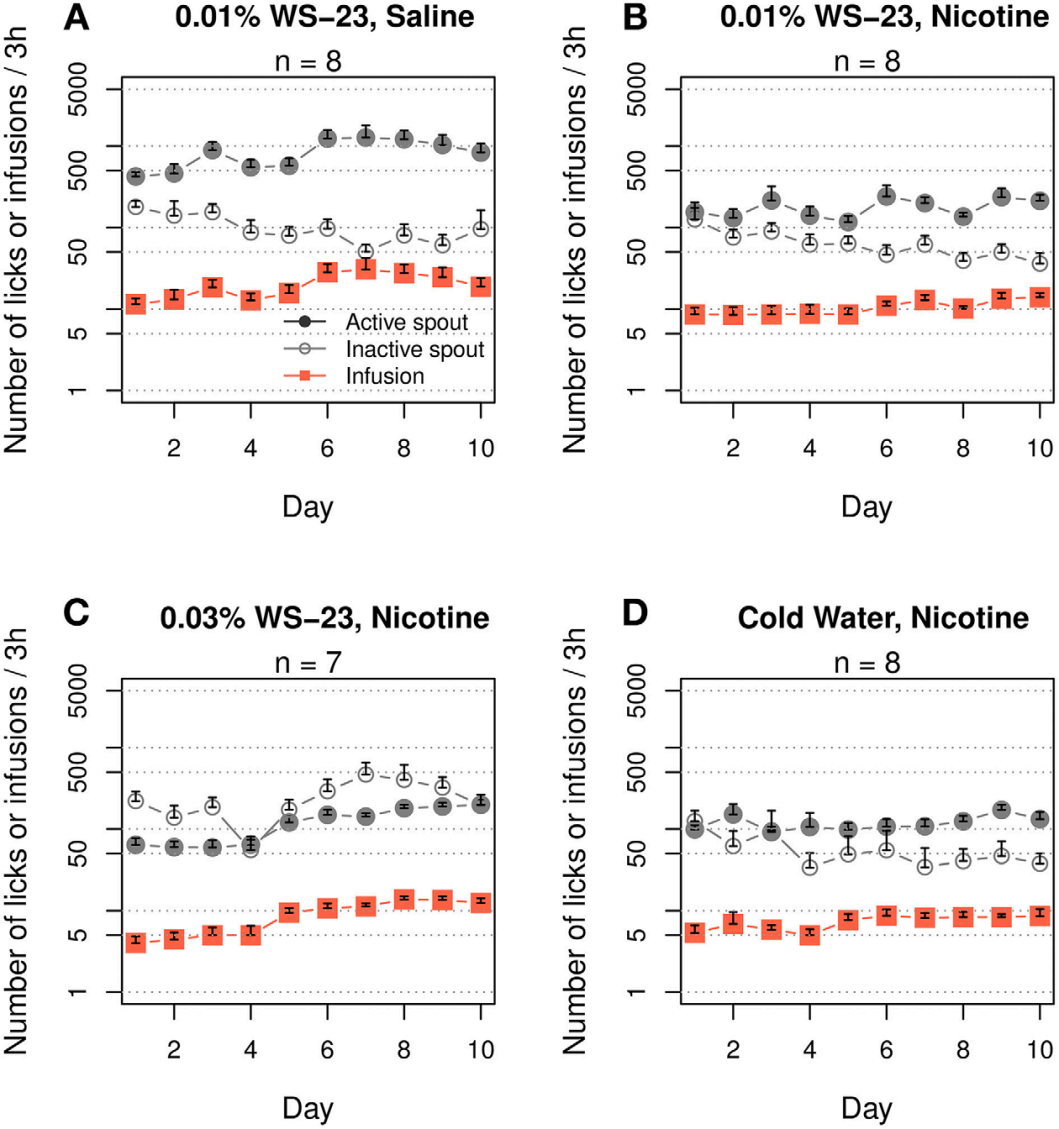
**Cooling sensation supported nicotine self-administration. (A)** WS-23 (0.01%) is an appetitive cue for i.v. saline self-administration. **(B)** WS-23 (0.01 %) induced significantly more active licks compared to inactive licks and resulted in a significant increase in the number of intravenous infusions across the sessions. **(C)** WS-23 (0.03%), which emits an odor, also supported increasing nicotine intake but induced more licks on the inactive spout than on the active spout. **(D)** Cold water (10.9°C) was effective in supporting nicotine self-administration.

We then tested whether WS-23 supports i.v. nicotine intake (Figure 4). The rats that self-administered saline with WS-23 as the cue exhibited a preference for the active spout (F_1, 90_ = 214.7, *p < 0.001*). The number of infusions did not significantly change across the sessions (F_9, 81_ = 1.6, *p* > 0.05). The rats that self-administered nicotine with 0.01% WS-23 as the cue exhibited a strong preference for the active spout (Figure 4B. F_1, 70_ = 89.0, *p* < 0.001). The number of infusions increased from 8.6 ± 1.7 in session 1 to 13.9 ± 1.7 in session 10 (effect of session: F_9, 63_ = 1.7, *p* > 0.05). The rats that self-administered nicotine with 0.03% WS-23, which had a detectable odor, increased the number of nicotine infusions from 4.0 ± 0.8 in session 1 to 12.4 ± 1.4 in session 10 (effect of session: F_9, 54_ = 11.4, *p* < 0.001). These two WS-23 groups had similar number of active licks (F_1, 13_ = 3.6, *p* > 0.05) and nicotine infusions (F_1, 13_ = 1.3, *p > 0.05*), but the high concentration group had significantly greater inactive licks than the low concentration group (F_1, 13_ = 7.8, *p* < 0.05).

To further confirm that the cooling sensation is a conditioned cue for nicotine reward, we tested cold water (~11°C) as the sensory cue. Similar to 0.01% WS-23, the use of cold water as a cue for nicotine IVSA resulted in a significantly greater number of active licks compared to inactive licks (Figure 4D, F_1, 64_ = 62.2, *p < 0.001*). Furthermore, the number of nicotine infusions tended to increase across the sessions (F_9, 54_ = 1.9, *p* = 0.07).

### 3.4. Composite cooling and olfactogustatory cue supports increasing i.v. nicotine intake

**Figure 5:**
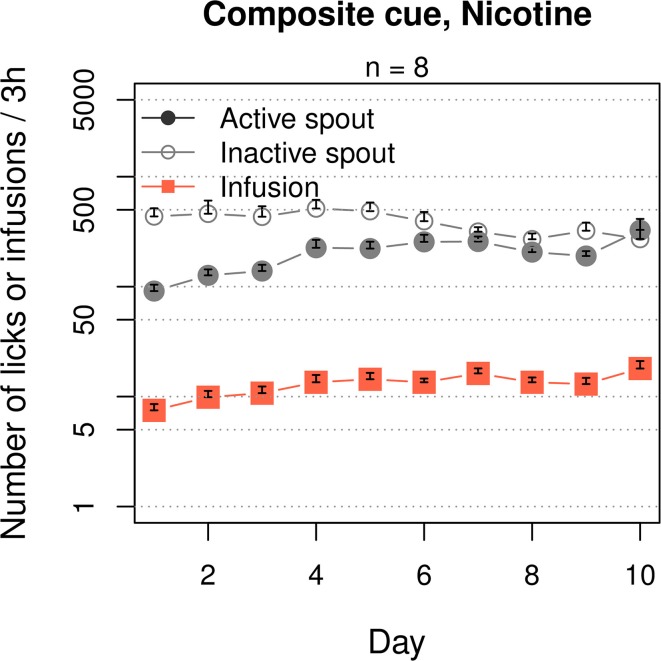
**A composite cue that induces cooling and olfactogustatory cues supported nicotine self-administration**. Similar to the groups that received an oral menthol cue (Figures 1, **9**), this group significantly increased the number of nicotine infusions across the 10 sessions, although more licks were emitted on the inactive spout compared to the active spout.

We then tested the effect of a composite cue that includes both cooling and olfactogustatory stimulations on nicotine IVSA (Figure 5). The rats received a composite cue that contained WS-23 (0.01%), saccharine (0.4%) and grape-flavored Kool-Aid® (0.1%). The rats licked more on the inactive spout compared to the active spout (F_1, 70_ = 39.1, *p* < 0.001). The numbers of active licks (F_9, 63_ = 4.7, *p* < 0.001) and nicotine infusions (F_9, 63_ = 5.6, *p* < 0.001) both significantly increased across the sessions. However, the number of inactive licks did not change (F_9, 63_ = 1.3, *p* > 0.05). On average, the rats obtained 14.8 ± 0.87 infusions during the last 3 sessions.

### 3.5. The ratio of active/inactive licks is an index of the affective value of the oral stimuli

**Figure 6:**
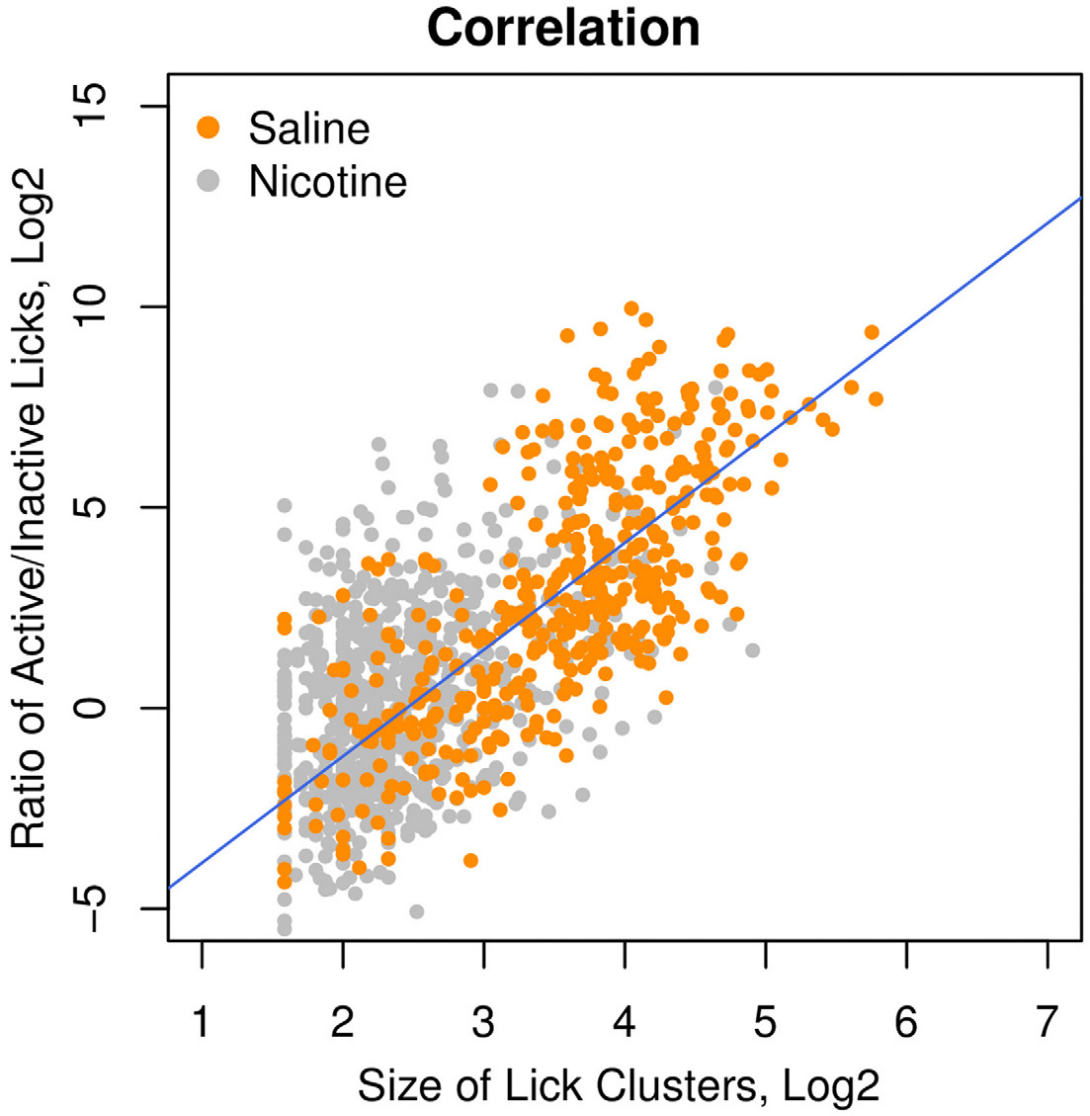
**The ratio of active/inactive licks was a measure of the affective value**. Among the rats that received different oral cues (i.e., menthol, grape flavor, and saccharin + glucose) and i.v. saline, the ratios of active/inactive licks were highly correlated (*r* = 0.75, *p* < 0.0001) with the size of the lick clusters, which was a measure of the affective value of oral stimuli. The correlation in the rats that received i.v. nicotine was also significant (*r* = 0.40, *p* < 0.0001).

Our previous study (Chen et al., 2011) suggested that an increased number of inactive licks is potentially associated with an aversive response to the stimuli. The affective value of the oral stimuli can be measured by the size of the lick clusters, defined as the number of licks within each round of licking (Davis and Smith, 1992). We therefore correlated the ratio of active/inactive licks with the size of the lick clusters and found a strong correlation (Figure 6, *r* = 0.75, *p* < 0.0001) among the rats that received i.v. saline with a variety of sensory cues (menthol, WS-23, saccharine + glucose, and grape flavor). One major advantage of the lick ratio over the cluster size is its wide dynamic range (2^5^–2^10^), which is several orders of magnitude greater than that of the lick cluster size (2^1^–2^5^). The wide dynamic range is especially advantageous when studying aversive stimuli, when the size of lick cluster is compressed to a very narrow range of 2–6 licks per cluster. A moderate, albeit highly significant, correlation (*r* = 0.40, *p* < 0.0001) between the lick ratio and lick cluster size was found in rats self-administering i.v. nicotine with these sensory cues. This reduced correlation in rats that self-administered nicotine is likely because nicotine reduced the size of the lick cluster to the lower end of its narrow dynamic range.

### 3.6. The interaction of the audiovisual cue and menthol

**Figure 7:**
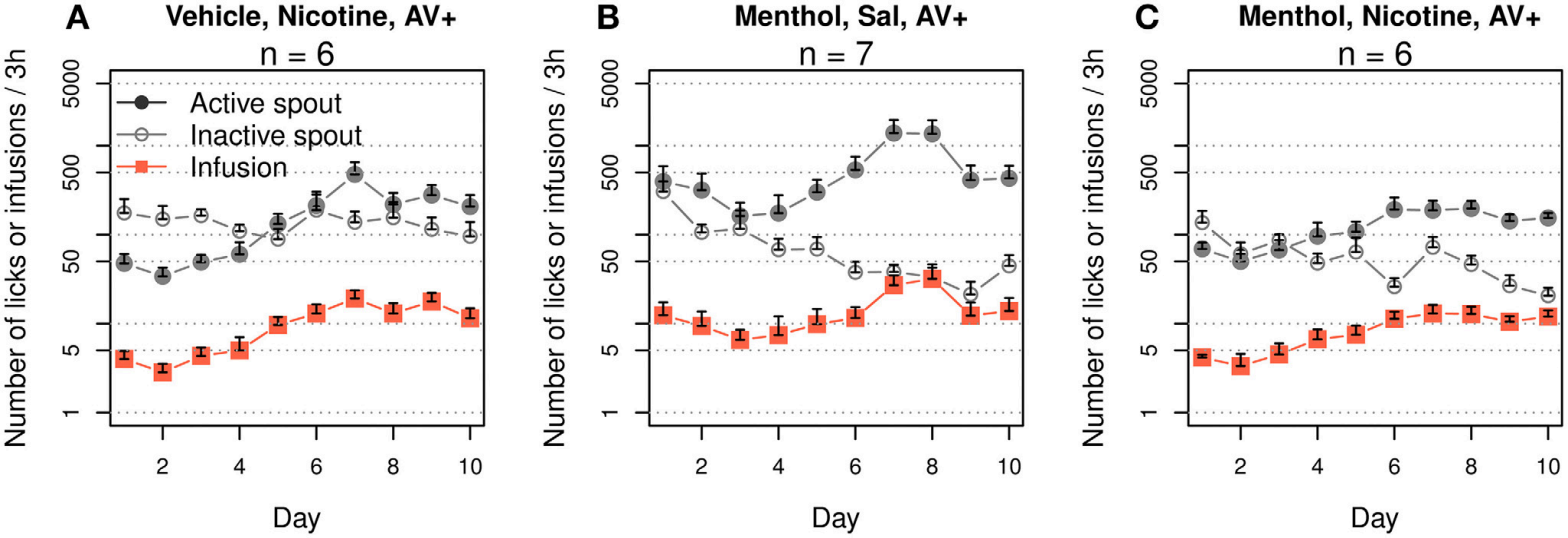
**The addition of an audiovisual (AV) cue enhanced the discrimination between the active and inactive spouts. (A)** Audiovisual cue enhanced nicotine self-administration with an oral vehicle cue (c.f. Figures 1E for the amount of active licks and figure 1A, Figure 8 for nicotine intake). There was a significant interaction between session and spout: the number of inactive licks was significantly higher than the number of active licks during the first five sessions but was significantly lower than the number of active licks during the last five sessions. **(B)** Audiovisual cue significantly enhanced the preference for the active spout in rats that self-administered i.v. saline with a menthol cue (c.f. Figure 1D). **(C)** Audiovisual cue significantly enhanced the preference for the active spout but not nicotine intake in rats that self-administered i.v. nicotine with a menthol cue.

We tested whether audiovisual cues could enhance the preference for the active spout when menthol was used as the contingent sensory cue for nicotine. We first tested the effect of an audiovisual cue on nicotine IVSA in rats received oral vehicle cue (Figure 7A). We found a significant interaction between the effect of the spout and that of the sessions (F_9, 50_ = 3.5, *p* < 0.01). There were fewer active licks than inactive licks for the first five sessions (F_1, 25_ = 19.4, *p* < 0.001), and the number of active licks was significantly greater than that of inactive licks for the subsequent five sessions (F_1, 25_ = 10.1, *p* < 0.01). The number of infusions significantly increased across the sessions (F_9, 45_ = 5.4, *p* < 0.001). On average, 3.7 ± 0.5 and 14.1 ± 1.9 infusions were obtained during the first and last three sessions, respectively. Compared to the group that self-administered nicotine with a vehicle cue but without an audiovisual cue (Figure 1E), the addition of an audiovisual cue had no effect on the number of inactive licks (F_1, 10_ = 2.5, *p* > 0.05) but significantly increased the numbers of active licks (F_1, 10_ = 6.5, *p* < 0.05) and nicotine infusions (F_1, 10_ = 8.4, *p < 0.05*).

A second control group received i.v. saline with a combined audiovisual and menthol cue (Figure 7B). The contingent audiovisual cue resulted in a preference for the active spout (F_1, 60_ = 46.9, *p* < 0.001). The number of infusions did not significantly change across the sessions (F_9, 45_ = 1.3, *p* > 0.05). Compared to rats that received the menthol cue without the audiovisual cue (Figure 1D), the audiovisual cue did not have a significant effect on the number of inactive licks (F_1,10_ = 2.6, *p* > 0.05) but significantly increased the numbers of active licks (F_1,10_ = 5.4, *p* < 0.05) and saline infusions (F_1, 10_ = 5.9, *p* < 0.05).

The rats preferred the active spout when i.v. nicotine was self-administered with a combined audiovisual and menthol cue (Figure 7C, F_1, 50_ = 41.8, *p* < 0.001). The effect of the session on the number of infusions was statistically significant (F_9,45_ = 3.3, *p* < 0.01). The number of infusions increased from 4.0 ± 0.35 during the first three sessions to 11.8 ± 0.68 during the last three sessions. Compared to the menthol-nicotine group without the audiovisual cue (Figure 1B), the audiovisual cue significantly reduced the number of inactive licks (F_1,10_ = 6.7, *p* < 0.05) but did not significantly change the number of active licks (F_1, 10_ = 0.42, *p* > 0.05) or nicotine infusions (F_1,10_ = 0.1, *p* > 0.05), indicating that the audiovisual cue enhanced the affective value of the complex stimuli that the rat received but not the amount of nicotine intake.

### 3.7. Comparison of nicotine infusions with different sensory cues

**Figure 8:**
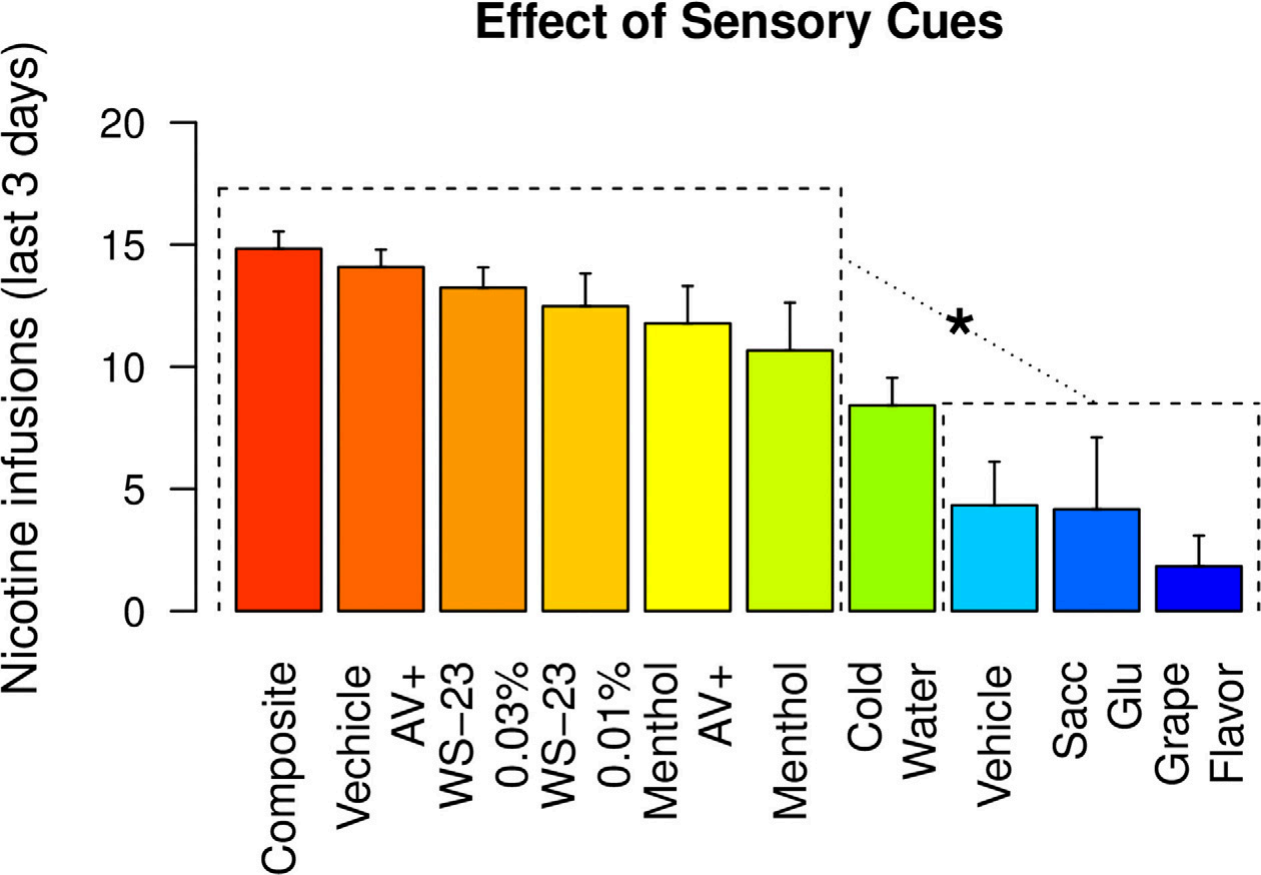
**The amount of nicotine intake with different sensory cues**. The amounts of nicotine infusions obtained during the last three IVSA sessions with 10 different sensory cues were plotted. Any of the six highest groups were statistically different from any of the three lowest groups. ^*^ *p* < 0.05, Tukey's HSD test.

Figure 8 compares the mean number of nicotine infusions self-administered by rats that received different sensory cues during the last three IVSA sessions. The rats that received menthol, WS-23, or audiovisual cues self-administered similar amounts of nicotine (Tukey's HSD, *ps* > 0.05). All of these groups obtained a significantly greater number of infusions compared to the groups that received vehicle, saccharin-glucose, or the grape-flavored cues (Tukey's HSD, *ps* < 0.001 to 0.05). The amount of nicotine intake by the cold water group was significantly greater than that of the grape-flavored group (Tukey's HSD, *p* < 0.05).

### 3.8. Extinction and reinstatement of nicotine-seeking behavior by a menthol cue

**Figure 9:**
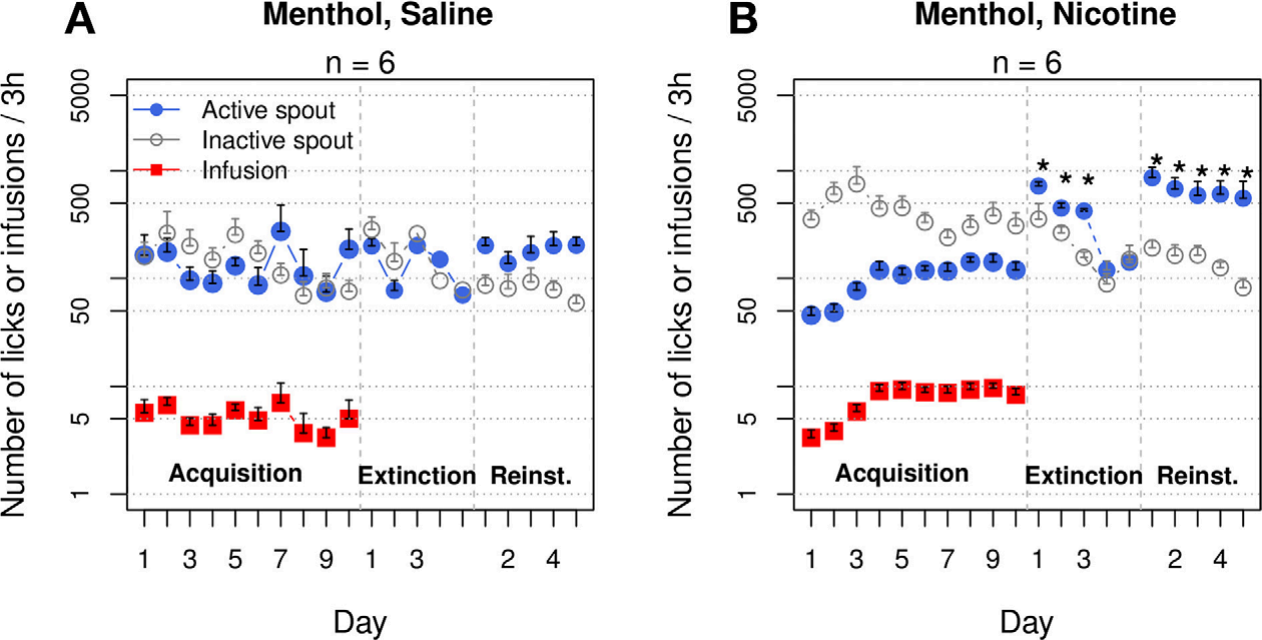
**Menthol induced an extinction burst and reinstated nicotine-seeking behavior**. The first two and last three extinction sessions are shown. **(A)** Rats that self-administered saline with a menthol cue reached the extinction criteria in 2.8 ± 0.8 sessions. The numbers of active spout licks during the first 2 days of extinction and during the five reinstatement sessions were not significantly different compared to those during the last 2 days of IVSA. **(B)** Rats that self-administered nicotine with a menthol cue increased the number of infusions across the sessions (*p* < 0.001), despite emitting significantly more licks on the inactive spout. Rats met the extinction criteria in 6.2 ± 0.8 sessions, which is significantly greater than the number of sessions for the saline rats (only one rat required a 5 day extinction period, *p* < 0.05). The number of active licks during the first three extinction sessions was significantly greater than that during the last IVSA session, indicating a strong extinction burst. Rats preferred the active spout during the reinstatement sessions (*p* < 0.001). The number of active licks was significantly greater during reinstatement than during the IVSA sessions and did not significantly change during the reinstatement tests. ^*^ *p* < 0.05 compared to the last session of IVSA.

Two additional groups of rats self-administered saline or nicotine with contingent menthol cues for 10 sessions (Figure 9). These groups replicated the majority of the results shown in Figures 1A,B. The rats in the menthol-saline group did not show a preference for either spout (F_1, 50_ = 1.4, *p* > 0.05), and the number of infusions did not significantly change across the sessions (F_9, 45_ = 1.4, *p* > 0.05). On average, very few infusions were obtained (4 ± 1.6). In contrast, the menthol-nicotine group increased the number of infusions across sessions (F_9, 45_ = 5.0, *p* < 0.001). The average number of infusions during the last three sessions was 9.1 ± 0.6. In contrast to the data shown in Figure 1B, in which the numbers of active and inactive licks were not different, the menthol-nicotine group exhibited a significantly greater number of inactive licks compared to active licks (F_1, 50_ = 57.5, *p* < 0.001).

The average number of sessions required to reach the extinction criteria was 2.8 ± 0.8 for the menthol-saline group, whereas the menthol-nicotine group required 6.2 ± 0.8 sessions (*p < 0.05)*. In the saline group, the number of active spout licks was not significantly different between the extinction and IVSA sessions (F_3, 15_ = 1.7, *p* > 0.05). In contrast, the nicotine group exhibited a significantly increased number of licks on the active spout during the extinction sessions (F_3, 15_ = 45.0, *p* < 0.001). The average number of licks on the active spout increased six-fold (from 119.5 ± 21.2 in the last IVSA session to 720.8 ± 63.5 in the first extinction session, *p* < 0.001, Tukey's HSD). During the second and third extinction sessions, the numbers of active spout licks in the nicotine group were 3.8–(*p* < 0.01, Tukey's HSD) and 3.5-fold (*p* < 0.05, Tukey's HSD) higher, respectively, than that in the last IVSA session.

The effect of menthol on drug-seeking behavior was tested over five consecutive sessions. Both groups displayed a preference for the active spout during the reinstatement sessions (F_1, 25_ = 16.4, *p* < 0.001 for the saline group and F_1, 25_ = 34.8, *p* < 0.001 for the nicotine group). The number of licks on the active spout during the reinstatement tests was significantly greater than that for the last five IVSA sessions in the nicotine group (F_9, 45_ = 4.5, *p* < 0.001), but not in the saline (F_9, 45_ = 0.6, *p* > 0.05) group. The number of active licks during the first reinstatement session was 862.0 ± 214.2, which was 7.2-fold greater than that during the last IVSA session in the nicotine group. On average, the number of active licks stayed within ~5-fold of that observed in the last IVSA session and did not significantly change during the reinstatement sessions (F_4, 20_ = 0.5, *p* > 0.05). Additionally, the number of active licks by the nicotine group during the reinstatement session was 3.6 ± 0.4-fold greater, on average, than that observed in the saline group (F_1, 10_ = 11.5, *p* < 0.01).

## 4. Discussion

Our main finding was that oral menthol as a sensory cue significantly facilitated nicotine IVSA in adolescent rats. This was demonstrated by an increased amount of self-administered i.v. nicotine in rats that received a menthol cue compared to those that received a vehicle cue or those that self-administered i.v. saline with a menthol cue. In addition, the rats that self-administered nicotine, but not saline, with a menthol cue exhibited significant extinction bursts and menthol-induced reinstatement of drug-seeking behavior. Together with the finding that yoked rats self-administered significantly less menthol compared to their masters, these data indicated that menthol is likely a conditioned cue for nicotine. Additional data showed that WS-23, a cooling compound, and cold water, though not two highly appetitive taste and odor cues, supported nicotine IVSA, indicating that the effect of menthol on the intake of nicotine is likely mediated by its cooling sensation.

Many potential mechanisms have been proposed to explain the effect of menthol on cigarette smoking. One hypothesis is that menthol facilitates the initiation of smoking by reducing the harshness of cigarette smoke via its anesthetic and cooling effects (Macpherson et al., 2006; Wise et al., 2011). This hypothesis predicts that menthol will increase the inhalation of cigarette smoke. However, clinical studies have found that menthol either decreases or has no effect on the puff frequency, where the puff volume and exhaled carbon monoxide results are conflicting or contradictory (Lawrence et al., 2011). A second potential mechanism is that menthol may modulate the metabolism of nicotine. For example, Benowitz et al. (2004) found that smoking menthol cigarettes inhibited the metabolism of nicotine in smokers by ~10% compared to non-menthol cigarettes. A third potential mechanism is that menthol may interact with nicotinic receptors. For example, menthol has been shown to inhibit the α4β2 (Hans et al., 2012) and α7 (Ashoor et al., 2013) nicotinic acetylcholine receptors. The behavioral consequence of this interaction has not yet been investigated.

It has been suggested that the sensory properties of menthol can serve as a conditioned reinforcer for nicotine. For example, Rose and Behm (2004) reported that the sensory attributes of menthol have a major influence on smoking reward. Ahijevych and Garrett (2010) also proposed that menthol may serve as a conditioned stimulus for nicotine. Our data are mostly in agreement with this hypothesis. We observed that when menthol was used as a contingent cue for nicotine, it increased the amount of the operant response to obtain nicotine compared to the vehicle cue and the menthol-saline controls (Figures 1A, 8). Furthermore, rats yoked to the menthol-nicotine masters, despite receiving the same amount of nicotine infusions, exhibited significantly less operant responses (Figures 1B,C). The requirement of contingent delivery of nicotine and a menthol cue supports the hypothesis that menthol functions as a conditioned cue for nicotine. This hypothesis also predicts that menthol will reinstate extinguished nicotine-seeking behavior, which is shown in Figure 9. In fact, menthol increased the number of active licks by ~5-fold throughout the five consecutive reinstatement tests in nicotine rats but had no effect on the number of licks in saline rats. Together, our data support the hypothesis that orally delivered menthol is a conditioned reinforcer for i.v. nicotine.

We analyzed the licking behavior of rats that received i.v. saline infusions with different olfactogustatory cues and found that the ratio of licks on the two spouts was highly correlated with the size of the lick clusters on the active spout (Figure 6), which is a reliable indicator of the affective value of oral stimuli and is not affected by satiety (de Araujo et al., 2006). Our data suggested that the lick ratio is a considerably more sensitive measure than the lick cluster size because it has a much wider dynamic range (Figure 6). Although we do not have a clear explanation as to why rats lick the inactive spout (attempting to wash away the bad taste?), interpreting the lick ratio as an indicator of the affective value is in agreement with the general sensory properties of the cues that we provided. For example, the saccharin/glucose solution is highly appetitive and has the highest lick ratio, whereas menthol is slightly aversive (Figure 1D) and induced more licks on the inactive spout only for the first few sessions, potentially because of habituation to its minor bitter taste (Green and Schullery, 2003). Furthermore, Figure 1F indicates that the vehicle (i.e., Tween 80) has an potential odor or taste that was appetitive, especially after repeated exposure. Consequently, similar to the other appetitive olfactogustatory cues (Figure 2), the vehicle failed to support nicotine IVSA (Figure 1E).

A cooling sensation is the main sensory component of menthol. The cooling sensation induced by either WS-23 (0.01%, Figure 4B) or cold water (~11°C, Figure 4D) as the cue supported nicotine IVSA with a strong preference for the active spout. These data indicated that similar to the audiovisual cue (Figure 7), the cooling sensation was also associated with the positive affective effect induced by nicotine. Slightly fewer infusions were obtained with the cold water cue compared to the menthol or WS-23 cues, potentially because the temperature of the water was not optimal or the stimulation did not last long enough. Olfactogustatory cues, however, were associated with the negative affective value induced by nicotine and did not support nicotine IVSA (Figures 2B,D). This result is consistent with previous findings that conditioned taste aversion is established between olfactogustatory cues and self-administered amphetamine (Wise et al., 1976) or nicotine (Chen et al., 2011), as well as the large body of literature on nicotine-induced conditioned taste aversion (Kumar et al., 1983). The differential association of cues with either positive or negative affective values induced by abused drugs in the same animal has previously been reported (Verendeev and Riley, 2011). Increasing the concentration of WS-23 produced a detectable odor (Figure 3), which resulted in a greater number of inactive licks (Figure 4C). Adding olfactogustatory components (i.e., saccharin and Kool-Aid®) to 0.01% WS-23 produced the same behavioral profile as 0.03% WS-23 (Figure 5). These data indicated that the increased number of inactive licks was caused by a nicotine contingent olfactogustatory cue. Rats that self-administered nicotine with the menthol cue exhibited the same behavioral profile (Figures 1B, 9) as these groups. This similarity indicated that the effect of menthol can be understood by its cooling and olfactogustatory effects: while the cooling sensation was associated with the positive affective effect of nicotine and supported nicotine IVSA, olfactogustatory stimulation, however, was associated with the negative affective effects of nicotine.

One puzzling aspect of the operant behavior of the menthol-nicotine group was that an increasing number of nicotine infusions was obtained despite the neutral or negative affective values (Figure 1B, 9). Furthermore, note that rats obtained similar amounts of stable nicotine intake when cooling sensations or audiovisual cues were present, regardless of the olfactogustatory cues used (Figure 8). Although these data seemingly suggested that the aversive properties of menthol (such as its bitter taste) conditioned with the aversive effect of nicotine to facilitate the licking behavior, a more likely mechanism is that the cooling sensation of menthol, which is appetitive, became a conditioned reinforcer for the reinforcing effect of nicotine. As discussed above, nicotine induces both reinforcing and aversive effects. The overall behavioral response induced by nicotine is greatly affected by its contingent cues. Positive cues associated with the reinforcing effects of nicotine, such as the cooling sensation, were not only required for the self-administration behavior but were also sufficient to drive the drug-taking behavior in the presence of cues that were associated with the negative effect of nicotine. This can be observed in the groups that exhibited sustained nicotine intake but not a preference for the active spout (e.g., menthol, 0.03% WS-23, and the composite cues), which can be understood as “wanting” nicotine but not “liking” it. There data are in agreement with clinical studies showing that nicotine is a substance with a strong addiction liability despite producing a minimal euphoric experience and is aversive during initial exposures (de Araujo et al., 2006).

Although nicotine IVSA has conventionally been studied using levers or nose poke holes as the manipulanda, Levin et al. (2010) and we (Chen et al., 2011; Wang et al., 2013) have shown that licking also supports operant nicotine IVSA. Overall, the menthol groups obtained ~10 infusions/3 h, which is lower than 10–15 infusions/1–2 h reported for the lever press method (Shaham et al., 1997; Kenny and Markou, 2006; Levin et al., 2011). However, the rats used in the short access lever press models were usually trained on food rewards, and some remained food-deprived during nicotine IVSA. Food deprivation is known to enhance drug reward (Carroll and Lac, 1993; Cabeza de Vaca and Carr, 1998). In contrast, rats obtained ~30–40 nicotine infusions (30 μg/kg) when they were trained 23 h/d without food deprivation (Valentine et al., 1997; O'Dell et al., 2006; Cohen et al., 2013). Considering that the majority of those infusions were obtained during the dark phase of the diurnal cycle, the rate of ~8–10 infusions per 3 h was almost identical to the data presented here. Therefore, the number of nicotine infusions obtained in our study is well within the expected range. Furthermore, the strong reinstatement of drug-seeking behavior (Figure 9) in the menthol-nicotine but not menthol-saline rats indicated that this amount of nicotine intake has significant behavioral consequences.

An extinction burst is characterized by a significant increase in operant response in animals undergoing initial extinction training. Extinction bursts have been observed for most abused drugs, including cocaine (Soria et al., 2008), heroin (Shalev et al., 2001), and ethanol (Lyness and Smith, 1992), and are thought to underlie the drug craving experienced by addicts during early withdrawal. Two reports have examined extinction bursts in nicotine IVSA in rats; neither found evidence of an extinction burst at the session level, although some rats showed an extinction burst during the peak response (Harris et al., 2007) or during the first 5 min of extinction (Pushparaj et al., 2012). In contrast, we found that the number of operant licks exhibited by the menthol-nicotine group increased six-fold compared with that in the last IVSA session. This drastic increase in response remained for the next two extinction sessions. In contrast, no extinction burst was found in the menthol-saline group (Figure 9). Furthermore, the number of licks on the previous active spout was ~2-fold greater than that on the inactive spout in the menthol-nicotine group during the first 2 days of extinction. The gradual reduction in the number of inactive licks is likely due to the removal of aversive stimuli. The different response patterns on the two spouts suggested that the association between the cooling sensation and the reinforcing effect of nicotine was considerably stronger than the association between the olfactogustatory stimuli and the aversive effect of nicotine. In addition to supporting the hypothesis that menthol is a conditioned reinforcer for nicotine, these results also suggested that smokers of menthol cigarettes are likely to experience a stronger craving for nicotine during withdrawal, which could result in lower smoking cessation rates (Okuyemi et al., 2007).

Menthol also induced strong drug-seeking behavior after extinction training in the menthol-nicotine rats (Figure 9). These rats emitted 5 − 7× more licks on the active spout compared with the last few IVSA sessions; no significant change in licking was observed in the menthol-saline rats. The elevated response remained stable throughout the five reinstatement sessions despite nicotine not being delivered. These results further strengthened the hypothesis that menthol gained reinforcing properties through its contingent presentations with nicotine during IVSA, thus becoming a conditioned reinforcer. These results are consistent with previous clinical studies that reported that menthol smokers had worse cessation outcomes than non-menthol smokers (Harris et al., 2004; Pletcher et al., 2006) and that menthol is likely a risk factor for relapse (Reitzel et al., 2013).

In summary, our data support the hypothesis that menthol contingently delivered with nicotine acquires reinforcing properties via a conditioning process. This effect is most likely attributable to the cooling sensation of menthol. We exclusively used female adolescent rats in this study. Whether the effect of menthol on nicotine self-administration differs based on the age and sex of the animals will be investigated in the future.

## Author contributions

Tengfei Wang contributed to the design of the experiments, collected data, conducted the initial data analysis, and drafted the first version of the manuscript; Bin Wang contributed to experimental design, data collection and data interpretation; and Hao Chen conceived the project, contributed to the design of the experiments, analyzed and interpreted the data, and revised the manuscript. All authors discussed the results and approved the final version of the manuscript.

## Funding

Funding was provided by an NIDA grant (DA-026894) and by the University of Tennessee Health Science Center awarded to Hao Chen.

### Conflict of interest statement

The authors declare that the research was conducted in the absence of any commercial or financial relationships that could be construed as a potential conflict of interest.
