# Supplementary material for: Menthol facilitates the intravenous self-administration of nicotine in rats
Source: Front Behav Neurosci. 2014 Dec 16;8:437. doi: 10.3389/fnbeh.2014.00437 (PMC4267270; doi:10.3389/fnbeh.2014.00437)
Supplement: Supplementary file 1 [file Image1.PDF]

Frontiers in Behavioral Neuroscience

Research Article
25 September 2014

Supplementary Material: Menthol facilitates the intravenous self-administration of nicotine in rats

Tengfei Wang [1], **Bin Wang** [2] **and Hao Chen** [1,*]

[1] *Department of Pharmacology, University of Tennessee Health Science Center, Memphis, TN, USA*
[2] *College of Pharmacy, Shaanxi University of Chinese Medicine, Xian Yang, Shaanxi, 712046. P.R. China.*

Correspondence*:
Hao Chen
Department of Pharmacology, University of Tennessee Health Science Center, 874 Union Ave. Memphis, TN, 38163, USA, hchen@uthsc.edu

1 SUPPLEMENTARY FIGURES

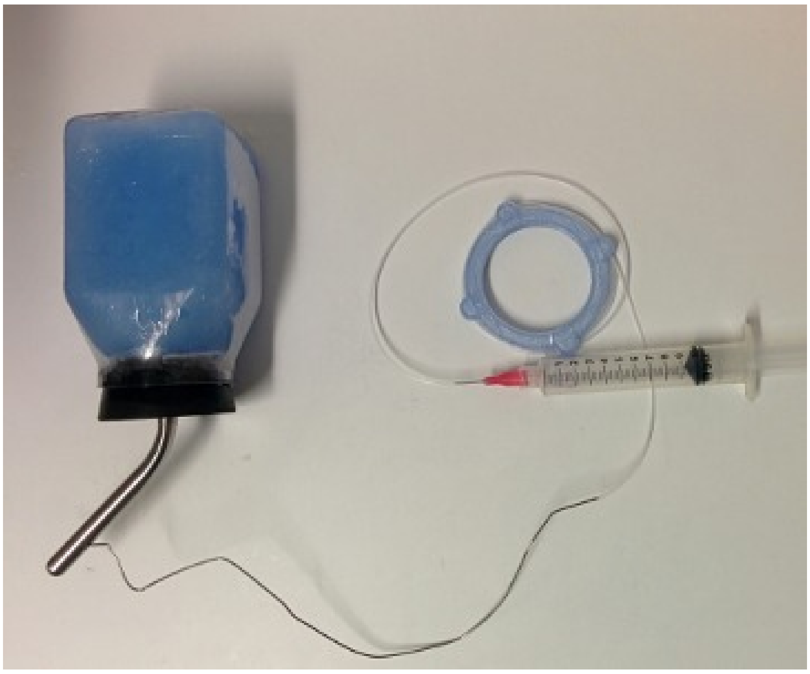

Supplementary Figure 1. A photograph of the cold water delivery system.

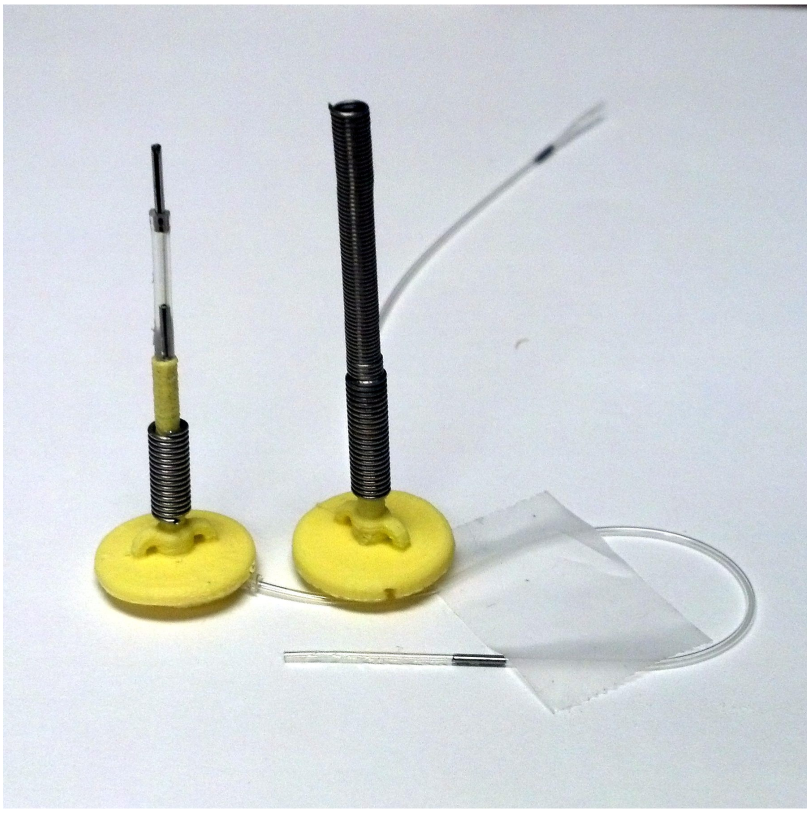

Supplementary Figure 2. A photograph of the assembled implant.
